# Beyond the Square knot: A validation study for a novel knot-tying method named “inverse 9”

**DOI:** 10.1016/j.heliyon.2023.e20673

**Published:** 2023-10-12

**Authors:** Xiangquan Qin, Ying Han, Yu Feng, Jiao Zhou, Siqi Guo, Tianfeng Xu, Dan Pu

**Affiliations:** a Department of West China Medical Simulation Center, West China Hospital of Sichuan University, Guoxue alley，Wuhou distrct, Chengdu, Sichuan Province, 610041, People's Republic of China; b Department of Breast and Thyroid Surgery, Southwest Hospital, the First Affiliated Hospital of the Army Military Medical University, Gaotanyan Street 29, Shapingba District, Chongqing, 400038, China

**Keywords:** Surgical knot, Laparoscopic surgery, Medical education, Minimal access surgery, Validation study

## Abstract

**Purpose:**

We compared the “inverse 9” laparoscopic suturing and knot-tying (LSKT) method to the traditional LSKT method in a validation study to demonstrate the “inverse 9” method's superiority and effectiveness in laparoscopy.

**Methods:**

On the basis of their experience in laparoscopic surgery, 78 trainees were divided into two groups, with 52 inexperienced trainees in group A and 26 experienced trainees in group B. In group A, 52 trainees were randomly allocated to either group A1 (“inverse 9” LSKT training) or group A2 (traditional LSKT training). In group B, experienced trainees were randomly assigned to receive “inverse 9” LSKT training (group B1) or continuing training in the traditional LSKT method (group B2). All trainees received the same instruction and assessment and were asked to provide a subjective assessment of the two training methods at the end of the training.

**Results:**

The trainees in groups A1, A2, and B had similar average ages and were mostly male. After training, all showed preliminary mastery of LSKT (*P* < 0.05). The trainees in groups A1 and B1 achieved learning proficiency in the fifth assessment, while those in group A2 achieved it in the sixth assessment. The trainees in groups A1 and B1 showed lower difficulty in achieving mastery and lower operation fatigue scores (*P* < 0.05), and 61.50 % of the trainees in group B preferred the “inverse 9” method in subjective evaluation.

**Conclusion:**

As a novel LSKT technique, “inverse 9” offers a multitude of benefits. In addition to ensuring a simpler operation and effectively reducing the knot-tying time, it also involves a shorter learning curve than traditional LSKT methods. As such, it can be easily mastered and widely adopted as a standard LSKT technique.

## 1 Introduction

Minimally invasive surgery has gained popularity with the widespread adoption of the concept and the advancements in laparoscopy, leading to the gradual replacement of open surgery [1]. In comparison with traditional open surgery, minimally invasive surgery offers benefits such as smaller incisions, reduced tissue damage, decreased postoperative pain, and shorter hospital stays [[2], [3], [4]]. However, minimally invasive surgery is associated with inherent risks, including complications related to anesthesia, bleeding, and infection [3,5]. The complexity of laparoscopic suturing and knot-tying (LSKT) is a contributing factor to the longer operating times associated with this procedure.

Unfortunately, the slow adoption of LSKT can be attributed, in part, to limitations with laparoscopic technology [6]. Learning the traditional LSKT technique in a two-dimensional environment, particularly with restricted visibility and limited angles, can be an arduous and frustrating experience for inexperienced surgeons [7]. Despite the availability of numerous LSKT techniques, many of them cannot ensure sufficient knot stability. Some surgeons have attempted to address this issue by using specialized knot-tying instruments [8], but these instruments can be expensive and have not yet demonstrated the same level of knot-tying efficacy as traditional surgical techniques [9]. The traditional LSKT technique has long been recognized as a challenging and time-consuming skill in laparoscopic surgery and is associated with a steep learning curve for inexperienced surgeons, so many surgeons believe that it causes operation fatigue [10]. Despite the availability of numerous laparoscopic knot-tying methods [11], most of them are difficult to operate and have prolonged knot-tying durations.

Based on the experience gained from previous LSKT, along with further exploration and analysis, the authors developed a new technique named the “inverse 9” method that incorporates the use of cavity mirrors for surgical exploration. The innovative “inverse 9” surgical knot-tying technique involves creating an “inverse 9” shape with the suture needle by utilizing the bending and shaping of the thread. Not only does this technique simplify the multiple steps involved in the traditional method of suturing, but it also saves operation time and may even address the issue faced by inexperienced surgeons in learning laparoscopic surgical skills.

Therefore, we conducted a validation study to compare the “inverse 9” LSKT method and the traditional LSKT method and thus evaluate the practicality and superiority of the former method.

## 2 Methods

### 2.1 Participants

A group of 78 trainees with specializations in general surgery, urology, obstetrics, gynecology, and other fields participated in this training program. Among the trainees, 26 had prior experience with LSKT through traditional laparoscopic surgery and had performed more than 30 laparoscopic surgeries, while the remaining 52 had no prior experience with LSKT. All trainees were required to complete the training content for each credit hour and undergo assessments to meet the program's training standards.

### 2.2 Equipment

The SR-R1 laparoscopic technique simulation trainer (SL-PE480C, Shiheng, Shanghai) was the primary tool used for training in this study. The training equipment consisted of a laparoscopic simulator and several instruments and modules, including two non-injury grasping forceps, two separation forceps, a tissue scissors, two needle holders, and a self-created suture module with 3-0/15-cm needle sutures. Besides, it includes a 5-megapixel USB camera capable of displaying a resolution of 1920 × 1080; the camera can be directly connected to a personal computer, which allows for direct recording using Windows camera software.

## 3 Tasks and measures

### 3.1 Experimental process and study protocol

#### 3.1.1 Inclusion criteria and grouping

The study included 78 trainees with a bachelor's degree or higher who participated in laparoscopic surgery skill training. Among the participants, 26 had prior experience with traditional LSKT under laparoscopic surgery, having performed more than 30 laparoscopic surgeries, and were assigned to group B. The remaining 52 trainees without prior LSKT experience were randomly divided into groups A1 (n = 26) and group A2 (n = 26). The participants in group A1 received training in the “inverse 9” LSKT method, while those in group A2 received training in the traditional LSKT method. The participants in group B1 continued learning the “inverse 9” LSKT method, building on their experience with the traditional LSKT method, while the participants in group B2 received continuing training in the traditional LSKT method.

#### 3.1.2 Explanation and guidance

During laparoscopic surgery skill training, the participants received thorough explanation of the technical details and key points regarding the two LSKT methods through video teaching. The trainees also received personalized guidance to help improve their skills.

#### 3.1.3 Training period

**Fig. 1:**
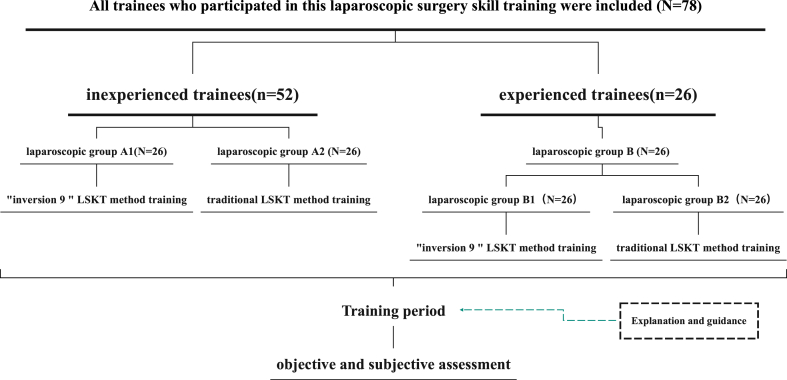
Flowchart of the study protocol.

During the training period, the participants completed eight rounds of concentrated training, with each round lasting approximately 45 min. The training content included information regarding the “inverse 9” LSKT method and the traditional LSKT method, and subjective evaluations were conducted using questionnaires after each round, resulting in a total of eight evaluations. Upon completing all rounds of training, the participants underwent objective and subjective evaluations using the evaluation criteria outlined in Chinese Laparoscopic Skills Testing and Assessment (CLSTA) [12]. The subjective assessments measured the perceived difficulty of mastering the two LSKT methods (on a scale of 0–10), operation fatigue (on a scale of 0–10), and the preferred knotting method (0, “inverse 9” LSKT method; 1, traditional LSKT method) as illustrated in Fig. 1.

#### 3.1.4 Technical highlights

##### 3.1.4.1 The “inverse 9” surgical knot-tying technique

The procedure for the “inverse 9” surgical knot-tying technique is as follows. Pass a needle through the surgical incision using a needle holder, and pull the suture to the midline of the incision with forceps, forming an “inverse 9″ shape between the suture and the needle exit. Pass the needle holder through the formed “inverse 9″ loop to turn for one round, thus completing two loops of suture. After clamping the end of the suture with the needle holder, pull the needle holder as well as the forceps that hold the suture in opposite directions at 180° to complete the first knot. Next, return the forceps that hold the suture to the center of the surgical incision to form an “inverse 9″ shape between the suture and the needle entry incision. Pass the needle holder through the formed “inverse 9″ loop to clamp the end of the suture, and pull the needle holder as well as the forceps that hold the suture in opposite directions at 180° to complete the second knot. Repeat the process for the second knot to finally complete the “inverse 9″ surgical knot-tying procedure under endoscopy. Refer to Fig. 2a and Video 1-2 for the details of the procedure.

**Fig. 2a and Video 1-2:**
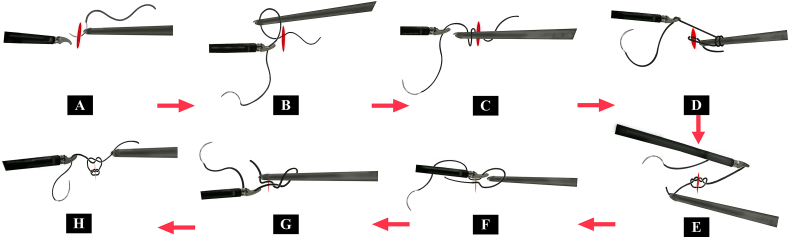
Steps in the “inverse 9” presurgical laparoscopic suturing and knot-tying method.

##### 3.1.4.2 Traditional surgical knot-tying method

The procedure for the traditional surgical knot-tying method is as follows. Pass a needle through the surgical incision using a needle holder. Pull the suture with forceps, leaving a certain length from the needle exit. Turn the needle holder around the suture pulled by the forceps to complete two loops of suture and clamp the end of the suture. Then, pull the needle holder as well as the forceps that hold the suture in opposite directions at 180° to complete the first knot. Continue to turn the needle holder around the suture for one round, and pull the needle holder as well as the forceps that hold the suture in opposite directions at 180° to complete the second knot. Repeat the process for the second knot to finally complete the traditional surgical knot-tying procedure under endoscopy. Refer to Fig. 2b and Video 3 for the details of the procedure.

**Fig. 2b and Video 3:**
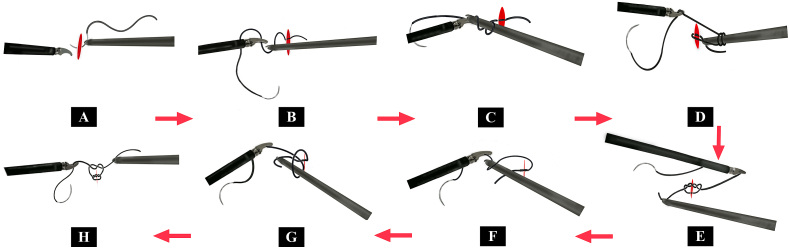
Steps in the Traditional laparoscopic suturing and knot-tying method.

##### 3.1.4.3 Statistical analysis

All data were processed with Microsoft Excel (Microsoft, Inc., USA). They were reported as mean ± SD for continuous variables and as the frequency (%) for categorical variables, and data were analyzed using the nonparametric Kruskal–Wallis test. *P* values < 0.05 were considered to indicate statistical significance; all tests were two-tailed. All statistical analyses were performed using SPSS 27 (SPSS Inc., Chicago, IL). Figures for the knot-tying time and the assessment score were generated using GraphPad Prism (version 9.2), Adobe Photoshop 2022, and Pixelmator Pro 2.2. In this study, we analyzed the learning curve for operation time using the cumulative sum (CUSUM) method [13], and all CUSUM analyses were performed with the statistical package “qcc” in R programming language (R version 4.0.2) [14].

##### 3.1.4.4 Ethical review and registration

All participants completed the relevant examinations after training and recorded their scores. The study was approved by the Biomedical Ethics Review Committee of Huaxi Hospital of Sichuan University (No. 1071/2019).

## 4 Results

### 4.1 Trainee characteristics

The average age of trainees was 34 years in groups A1 and A2 and 36 years in group B. More than half of the trainees in all the groups were male. In group A1, 50 % of the trainees were engaged in general surgery. Additionally, over half of the training participants had a bachelor's degree. Groups A1, A2, and B showed no significant differences in baseline characteristics (*P* > 0.05). Other details are listed in Table I.

**Table 1:** Demographic characteristics of the trainees in the three groups.

Variable^a^	Group A1	Group A2	Group B	*P-value*
Age (years)	34.23 ± 3.42	34.54 ± 5.54	36.12 ± 4.84	0.61
Sex				0.47
Male	17 (65.4 %)	19 (73.1 %)	21 (80.8 %)	
Female	9 (34.6 %)	7 (26.9 %)	5 (19.2 %)	
Qualification				0.98
Undergraduate	19 (73.1 %)	18 (69.2 %)	24 (92.3 %)	
Postgraduate	6 (23.1 %)	7 (26.9 %)	2 (7.7 %)	
PhD	1 (3.8 %)	1 (3.8 %)	0	
Professional Titles				0.21
Resident	3 (11.5 %)	2 (7.7 %)	2 (7.7 %)	
Attending Physician	22 (84.6 %)	20 (76.9 %)	18 (69.2 %)	
Deputy Chief Physician	1 (3.8 %)	4 (15.4 %)	6 (23.1 %)	
Department				0.39
General Surgery	13 (50.0 %)	10 (38.5 %)	10 (38.5 %)	
Hepatobiliary Surgery	1 (3.8 %)	2 (7.7 %)	4 (15.4 %)	
Urology	8 (30.8 %)	4 (15.4 %)	6 (23.1 %)	
Thoracic Surgery	2 (7.7 %)	4 (15.4 %)	2 (7.7 %)	
Gynecology and Obstetrics	2 (7.7 %)	6 (23.1 %)	4 (15.4 %)	

a Data are presented as mean*±* SD or number (%).

### 4.2 Objective assessments and evaluations after all eight rounds

We collected the data from all eight subjective trainee evaluations from groups A1, A2, B1, and B2. Among these, 12 trainees failed to undergo examinations on time due to absence from work, and their initial examination results were recorded as null values.

In the first evaluation, group A2 had the lowest scores and longest times for suture time, knotting time, total knot-tying time, and knot-tying time score, followed by group A1. In contrast, group B2 had the highest scores and shortest times. However, over the subsequent evaluations, the knot-tying time of groups A1, A2, and B1 gradually decreased. Among them, group A1 showed the most significant improvement, with the shortest knot-tying time consistently lower than those of groups A2 and B1/B2. On the other hand, the knot-tying time of group B2 did not show a significant change over time.

Similarly, the suture time of groups A1 and A2 also gradually decreased over the course of the eight evaluations, while the suture time of group B1/B2 remained relatively stable. The total knot-tying time of all groups also decreased over time, with group A1 showing the most significant improvement.

**Fig. 3:**
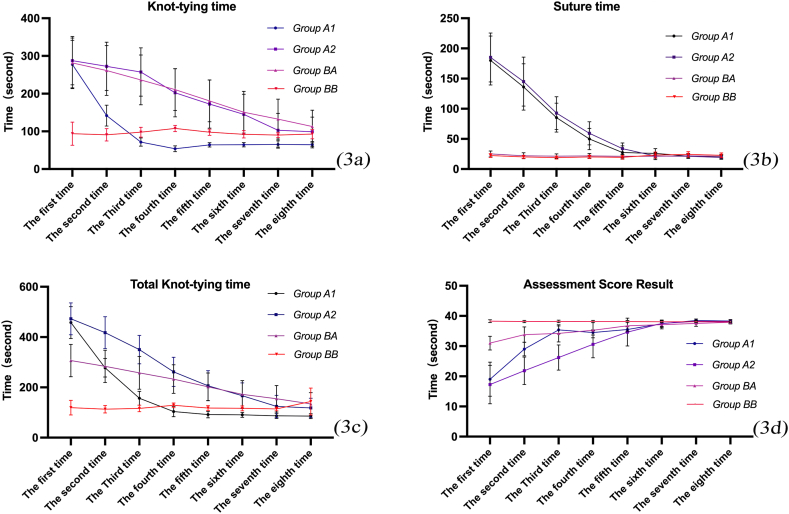
(a) Knot-tying time for all trainees; (b) suture time for all trainees; (c) total knot-tying time for all trainees; (d) assessment scores for all trainees.

In terms of assessment scores, group B2 consistently scored the highest throughout the assessments, with no significant changes over time. In contrast, groups A1, A2, and B1 had lower scores at the beginning, but their scores gradually increased over time, approaching and even exceeding the scores of group B2 (Fig. 3a–d).

### 4.3 Learning curve analysis

After analyzing the data from eight assessments conducted among the three groups, we observed a significant change in knot-tying time in groups A1, A2, and B1 during the assessment period. All three groups were learning a new laparoscopic knot-tying method, either the “inverse 9” LSKT method or the traditional LSKT method, indicating the involvement of a learning period.

To assess the impact of case experience accumulation on knot-tying time for both the “inverse 9” LSKT method and the traditional LSKT method during training, we analyzed the overall knot-tying time and the time spent for docking using the CUSUM method to analyze the learning curve. A learning curve was considered complete when a point indicating a decrease in knot-tying time was observed on the CUSUM plot.

**Fig. 4:**
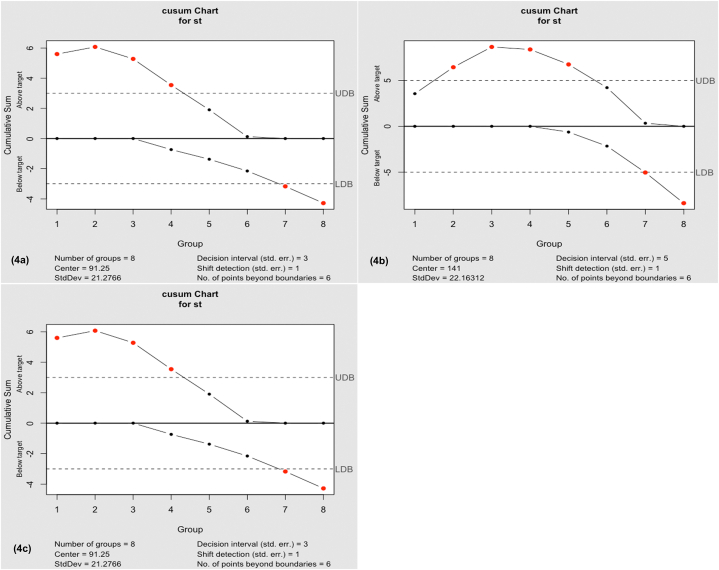
(a) Knot-tying time and learning curve in group A1: The docking time for 26 trainees who received training in the “inverse 9” laparoscopic suturing and knot-tying (LSKT) method. The cumulative sum (CUSUM) curve demonstrated that docking time improved from the fifth round and continued to improve.(b) Knot-tying time and learning curve in group A2: The docking time for 26 trainees who received training in the traditional LSKT method. The CUSUM curve demonstrated that docking time improved from the sixth round and continued to improve.(c) Knot-tying time and learning curve in group B: The docking time for 26 trainees who received training in the “inverse 9” LSKT method. The CUSUM curve demonstrated that docking time improved from the fifth round and continued to improve.

Based on the CUSUM graph, we collected and analyzed the knot-tying time from eight evaluations conducted in groups A1, A2, and group B1. Group A1/B1 completed their learning of the “inverse 9” LSKT method and achieved proficiency in the fifth examination and evaluation. Group A2, on the other hand, completed learning of the traditional LSKT method and achieved proficiency in the sixth examination and evaluation (Fig. 4a–c).

### 4.4 Findings from the seventh and eighth objective assessments and evaluations

After analyzing the learning curves, we found that all trainees in groups A1, A2, and B1 achieved proficiency in the seventh and eighth examinations and assessments. Therefore, we compared the results of the seventh and eighth assessments to evaluate the progress made.

The knotting time, total knot-tying time, and knot-tying score differed significantly between groups A1 and A2 in the 7th and 8th assessments (*P* < 0.05). The knot-tying times for group A1 in the 7th and 8th assessments were 65.03 ± 9.66 s and 64.65 ± 8.47 s, respectively, while the overall knot-tying times were 86.65 ± 10.32 s and 85.57 ± 9.81 s, respectively. The knot-tying score for the seventh and eighth assessments was 16.00 points. The B1/B2 groups also showed significant differences in the knotting time, total knot-tying time, and knot-tying score in the seventh and eighth assessments (*P* < 0.05). The knot-tying times for group B2 in the seventh and eighth assessments were 90.15 ± 6.50 s and 92.84 ± 12.57 s, while the total knot-tying times were 114.38 ± 7.69 s and 115.62 ± 11.88 s, respectively. The knot-tying score for the seventh and eighth assessments was 16.00 points. Furthermore, we found no significant difference in quality and knot-tying score among group A1, group A2, and group B1/B2 trainees (*P* > 0.05; Table II).

**Table 2:** Comparison of the results of the seventh and eighth assessments of trainees in the three groups.

Variable^a^	Group A1	Group A2	*P*_*1*_	Group B		*P*_*2*_
				Group B1	Group B2	
Suture time 7th (s)	21.61 ± 4.08	21.15 ± 3.27	0.66	22.07 ± 2.86	24.23 ± 4.63	*0.05*
Knot-tying time 7th (s)	65.03 ± 9.66	102.88 ± 44.80	<0.001	132.50 ± 53.01	90.15 ± 6.50	< *0.001*
Total time 7th (s)	86.65 ± 10.32	124.04 ± 44.29	<0.001	154.57 ± 52.59	114.38 ± 7.69	< *0.001*
Score for knot-tying time 7th (points)	16.00 ± 0.00	15.81 ± 0.49	0.045	15.46 ± 0.94	16.00 ± 0.00	*0.01*
Score of knot-tying quality 7th (points)	22.46 ± 0.51	22.38 ± 0.49	0.58	22.08 ± 0.27	22.08 ± 0.27	*1.00*
Total score 7th (points)	38.46 ± 0.51	38.19 ± 0.75	0.14	37.54 ± 0.98	38.08 ± 0.27	*0.1*
Suture time 78th (s)	20.92 ± 3.15	19.31 ± 3.11	0.07	21.23 ± 3.01	22.76 ± 3.98	*0.12*
Knot-tying time 8th (s)	64.65 ± 8.47	99.04 ± 38.79	< *0.001*	112.92 ± 43.38	92.84 ± 12.57	*0.03*
Total time 8th (s)	85.57 ± 9.81	118.35 ± 38.41	< *0.001*	134.15 ± 44.39	115.62 ± 11.88	*0.045*
Score for knot-tying time 8th (points)	16.00 ± 0.00	15.85 ± 0.37	0.04	15.81 ± 0.40	16.00 ± 0.00	*0.02*
Score for knot-tying quality 8th (points)	22.30 ± 0.47	22.35 ± 0.49	0.77	22.08 ± 0.27	22.04 ± 0.19	*0.56*
Total score 8th (points)	38.31 ± 0.47	38.19 ± 0.63	0.46	37.88 ± 0.52	38.04 ± 0.19	*0.16*

a Data are presented as mean*±*SD or number (%).

### 4.5 Subjective assessment and evaluation

Subjective evaluation and feedback assessments were performed after all trainees completed their learning and assessment. Among the 78 trainees, five from group A1 and three from group A2 did not participate in the training feedback evaluation and were marked as null. All participants in group B completed the training feedback evaluation.

The difficulty score for mastering the skill was lower in group A1 (2.46 ± 0.51 points) than in group A2. The operation fatigue score for group A1 was also lower (1.81 ± 0.63 points). The comparison between groups A1 and A2 showed significant differences (*P* < 0.001). The operation fatigue score for group B1 was 2.15 ± 0.54 points, which was significantly different from that of group B2 (*P* < 0.001).

In terms of feedback on the final selection of the best knot-tying method, 61.50 % of trainees in group B preferred the “inverse 9” LSKT method, while 38.50 % preferred the traditional LSKT method. However, the *P*-value for this comparison was 0.24 and did not indicate a significant difference in preference (Table 3).

**Table 3:** Subjective assessment and evaluation in three groups.

Variable^a^	Group A1	Group A2	*P*_1_	Group B		*P*_2_
				Group B1	Group B2	
Degree of difficulty (points)	2.46 ± 0.51	3.23 ± 0.59	< *0.001*	2.77 ± 0.51	2.92 ± 0.63	0.34
Operation fatigue score (points)	1.81 ± 0.63	3.46 ± 0.58	< *0.001*	2.15 ± 0.54	3.42 ± 0.50	< *0.001*
Optimal selection method for knot-tying				16 (61.50 %)	10 (38.50 %)	0.24

a Data are presented as mean*±*SD or number (%).

## 5 Discussion

This is the first report on our laparoscopic suturing and knot-tying method, known as the “inverse 9” technique. This method breaks away from the conventional steps and procedures of laparoscopic suturing and knot-tying. Through a randomized controlled validation study, it has been demonstrated that this innovative technique can optimize and enhance the efficiency of suturing and knot-tying in laparoscopic procedures, reducing the level of difficulty and making it more accessible for beginners.

The results obtained after eight credit hours of training and assessment showed that among trainees without laparoscopic operation experience, as training and examinations progressed, the knot-tying time and quality of the “inverse 9” LSKT method improved and even surpassed the traditional LSKT method in objective assessment and evaluation scores. Thus, in addition to being comparable to the traditional LSKT method widely used in clinical practice, the “inverse 9” LSKT method for laparoscopic knot-tying training can also further shorten knot-tying time and improve efficiency under laparoscopy. We attribute this mainly to the following two factors: 1) We have streamlined the suturing technique to eliminate unnecessary or redundant steps, resulting in a more efficient process. 2) Through practice and experience, we have enhanced our manual dexterity, allowing for faster and more precise suturing and knot-tying.

We also compared the proficiency levels required for the two methods by using the CUSUM method to analyze the learning curves of eight knot-tying examinations. The results demonstrated that the trainees using the “inverse 9” LSKT method achieved proficiency faster than those using the traditional LSKT method, confirming that the “inverse 9” LSKT method can be mastered more quickly. The traditional LSKT method's laparoscopic winding operation was found to increase the knot-tying time due to its difficulty and uncertainty, as reported previously in the literature [15]. Additionally, the coiling operation in the traditional LSKT method required a longer thread length and more operating space [16,17], which was not necessary in the “inverse 9” LSKT method. This expanded the range of thread length adaptability, allowing the “inverse 9” LSKT method to be used for both long and short threads in laparoscopic suturing and knot-tying.

To further compare the effectiveness of the two methods, we analyzed their learning curves and eliminated the results from the first six assessments to avoid the impact of some learners not fully mastering the methods during the initial learning period. We then compared the knot-tying time, knot-tying quality, and assessment scores from the seventh and eighth objective assessments. Our analysis showed no significant differences in knot-tying quality or assessment scores between the two methods. However, the knot-tying time was notably shorter and the assessment score was higher for the “inverse 9” LSKT method, even when it was used by inexperienced surgeons or those with limited experience in cavity mirror operations. Thus, the “inverse 9” LSKT method shows some advantages over other methods in terms of knot-tying time, and this benefit may extend to inexperienced surgeons as well. Finally, our subjective evaluation of the difficulty and operation fatigue among all learners showed that the subjective assessment score when using the “inverse 9” LSKT method was lower than that obtained using the traditional LSKT method, which was consistent with our expected results.

In addition, 61.5 % of surgeons with some experience in laparoscopic operation were willing to choose the “inverse 9” LSKT method, while 38.50 % of surgeons chose the traditional LSKT method, although there was no significant difference (*P* > 0.05). This could have occurred because some surgeons with a certain level of experience in laparoscopic operations are accustomed to the traditional LSKT method, so they are not willing to change their previous operating technique [18].

In the three groups, most trainees who participated in laparoscopic training were primarily males, and primarily engaged in general surgery, which were consistent with previous relevant reports [19], indicating the widespread adoption of laparoscopic technology with advancements in materials science and the increasing interest among young surgeons to enhance their endoscopic or laparoscopic skills through training. This trend is expected to further promote the growth and dissemination of minimally invasive surgery in the future.

However, our study had several limitations. First, the sample size in each group was relatively small. Second, the clinical information collected for the trainees was incomplete. For instance, we only collected information regarding the number of laparoscopic surgeries performed by trainees in the past, without specifying their suturing and knot-tying performance during laparoscopic surgery. Thus, past incorrect surgical habits may have had an impact on the effectiveness of training. Third, due to limited resources, we did not conduct a comparative study on different suture materials and different sizes of the operating space. Fourth, since clinical validation was not conducted, the results lacked reliability. In the future, conducting this study with experienced laparoscopic surgeons and clinical validation could yield more reliable data. Therefore, we aim to further verify the practicality and superiority of the two training methods in future clinical studies, including the use of different suture materials and operating space sizes.

## 6 Conclusion

In conclusion, the “inverse 9” LSKT method allows fast knot-tying and has a short learning curve, facilitating quick and skillful mastery. This makes it an attractive alternative with potential clinical applications for laparoscopic suture and knot-tying procedures.

## Funding

This study was supported by Postgraduate Education and Teaching Reform Research Project of 10.13039/501100004912Sichuan University (No. GSSCU2021150); Medical Simulation Research Project of National Medical Education Development Center (No. 2021MNYB07).

## Ethics statement

This research adheres to the ethical guidelines set forth by [HELIYON]. We have carefully reviewed the ethics declarations list provided by [HELIYON], available at [https://www.cell.com/heliyon/ethics], and have included all applicable statements in our ethical considerations for this manuscript.

All necessary ethical approvals, including institutional review board (IRB) or ethics committee approvals, were obtained prior to conducting this research. Reference numbers and approval dates are available upon request. Informed consent was obtained from all participants involved in this study. Participants were provided with detailed information about the study's purpose, procedures, potential risks, and benefits, and they voluntarily provided written consent before participating.

The authors declare no conflicts of interest related to this research. This study was conducted impartially and without any external influence that could affect the integrity of the findings. Data collection and analysis were performed following rigorous and appropriate methodologies. Data were collected with the highest standards of accuracy, and statistical analyses were conducted transparently to ensure the validity of the results.

Authorship of this manuscript accurately reflects the contributions of each author to the research. All authors have reviewed and approved the final version of the manuscript for submission. This manuscript is an original work, and proper credit has been given to the works of others through appropriate citations and references. No part of this manuscript has been plagiarized from other sources. This research respects the rights and welfare of human subjects. Any potential risks to participants were minimized, and necessary precautions were taken to protect their privacy and confidentiality. We are committed to upholding the highest standards of ethical conduct in our research. If you have any further inquiries regarding the ethical aspects of this manuscript, please do not hesitate to contact us.

## Data availability statement

The primary data included in supplementary Material in article. Supplementary materials containing additional datasets and analysis scripts are available as Supplementary Material Section.

## CRediT authorship contribution statement

**Xiangquan Qin:** Conceptualization, Data curation, Formal analysis, Investigation, Methodology, Project administration, Resources, Software, Supervision, Validation, Writing – original draft, Writing – review & editing. **Ying Han:** Conceptualization, Data curation, Formal analysis, Investigation, Methodology, Project administration, Resources, Writing – original draft, Writing – review & editing. **Yu Feng:** Data curation, Software. **Jiao Zhou:** Visualization. **Siqi Guo:** Data curation, Software. **Tianfeng Xu:** Data curation, Investigation. **Dan Pu:** Data curation, Formal analysis, Funding acquisition, Investigation, Project administration, Resources, Validation, Writing – review & editing.

## Declaration of competing interest

Xiangquan Qin, Ying Han, Yu Feng, Jiao Zhou, Siqi Guo, Tianfeng Xu, and Dan Pu have no conflicts of interest or financial ties to disclose.
